# Mitochondria-localized lncRNA HITT inhibits fusion by attenuating formation of mitofusin-2 homotypic or heterotypic complexes

**DOI:** 10.1016/j.jbc.2022.102825

**Published:** 2022-12-23

**Authors:** Xingwen Wang, Yi Zhang, Qingyu Lin, Kunming Zhao, Dantong Zhu, Ying Hu

**Affiliations:** School of Life Science and Technology, Harbin Institute of Technology, Harbin, Heilongjiang Province, China

**Keywords:** LINC00637, mitochondrial dynamics, MFN2, apoptosis, mitochondrial fusion, CLIP, cross-linking and immunoprecipitation, ER, endoplasmic reticulum, GST, glutathione S-transferase, HITT, HIF-1α inhibitor at translation level, HSP60, heat shock protein 60, IP, immunoprecipitation, KD, knockdown, lncRNAs, long noncoding RNAs, MFN, mitofusin, OPA, optic atrophy type, qRT-PCR, quantitative real time-PCR

## Abstract

Long noncoding RNAs (lncRNAs) are emerging as essential players in multiple biological processes. Mitochondrial dynamics, comprising the continuous cycle of fission and fusion, are required for healthy mitochondria that function properly. Despite long-term recognition of its significance in cell-fate control, the mechanism underlying mitochondrial fusion is not completely understood, particularly regarding the involvement of lncRNAs. Here, we show that the lncRNA HITT (HIF-1α inhibitor at translation level) can specifically localize in mitochondria. Cells expressing higher levels of HITT contain fragmented mitochondria. Conversely, we show that HITT knockdown cells have more tubular mitochondria than is present in control cells. Mechanistically, we demonstrate HITT directly binds mitofusin-2 (MFN2), a core component that mediates mitochondrial outer membrane fusion, by the *in vitro* RNA pull-down and UV-cross-linking RNA-IP assays. In doing so, we found HITT disturbs MFN2 homotypic or heterotypic complex formation, attenuating mitochondrial fusion. Under stress conditions, such as ultraviolet radiation, we in addition show HITT stability increases as a consequence of MiR-205 downregulation, inhibiting MFN2-mediated fusion and leading to apoptosis. Overall, our data provide significant insights into the roles of organelle (mitochondria)-specific resident lncRNAs in regulating mitochondrial fusion and also reveal how such a mechanism controls cellular sensitivity to UV radiation-induced apoptosis.

Mitochondria are the primary energy source for cells and are key platforms for signal transduction cascades involved in multiple biological processes, such as metabolism, migration, differentiation, senescence, and death (1, 2). Mitochondrial functions are tightly coupled to the dynamic networks that are balanced by flux between mitochondrial fusion and fission events (3). A high fusion-to-fission ratio results in long, tubular mitochondria, while a low ratio leads to fragmented mitochondria that look like spheres or short rods. Mitochondrial fusion is coordinately controlled by mitochondrial outer membrane protein mitofusin (MFN)1/2 and inner membrane protein optic atrophy type 1 (OPA1) (4). MFN1/2 functions by forming homotypic or heterotypic complexes at two closely adjacent mitochondria that subsequently link them together to initiate mitochondrial outer membrane fusion, while OPA1 mediates fusion of inner membranes (5). Genetic mutations in MFN2 suppress mitochondrial fusion and cause Charcot Marie Tooth Disease 2A and also cause the neurodegenerative disease (6, 7).

The balance of mitochondrial dynamics can be disrupted under multiple types of stress (8). For example, cells subjected to UV radiation or general inhibition of protein or mRNA synthesis have elongated mitochondria, which is driven by increased fusion and has been termed stress-induced mitochondrial hyperfusion (9). This event is essential for energy production and stress adaptation (8). Cells defective in mitochondrial fusion are vulnerable to stress-induced apoptosis (10, 11). It has also been shown that mitochondrial elongation and subsequent adaptation occur in response to stresses such as serum (12, 13) and amino-acid starvation (14, 15). Unlike hyperfusion, this is caused by decreased fission. However, although it is clear that mitochondrial fusion and fission are fundamental for mitochondrial functioning, the regulatory mechanisms underlying mitochondrial dynamics are not well understood.

Long noncoding RNAs (lncRNAs) are defined as RNA transcripts longer than 200 nt in length with no protein-coding potential (16). LncRNA genes, like many other noncoding genes, were once regarded as ‘genomic junk’ (17). However, over the past decade, it has been shown that lncRNAs are widely expressed and involved in almost every aspect of biological processes such as cell migration, metabolism, cell death, and differentiation (18, 19, 20). The altered expression of lncRNAs is strongly associated with a variety of diseases, including cancer (21, 22). LncRNAs can elicit either oncogenic or tumor-suppressive functions through specific interactions with DNA, RNA, or proteins at particular subcellular localizations (16, 18). Intriguingly, the development of fractionation-based techniques has shown that, in addition to their overall distribution in the nucleus or cytoplasm, organelle-specific lncRNAs exist in mitochondria (23), the endoplasmic reticulum (ER) (24), and nuclear paraspeckles (25). However, only a small proportion of organelle-associated lncRNAs have been fully validated or functionally characterized to date.

Recently, we characterized an lncRNA called HITT (HIF-1α inhibitor at translation level; also known as LINC00637). Decreased HITT expression are seen in multiple types of cancer and are associated with poor outcomes of patients, including colon and lung cancers. HITT can localize to both the nucleus and cytoplasm (26). In the cytoplasm, it binds to the translational regulator Y box–binding protein 1 and prevents it from binding to its target, HIF-1α mRNA, resulting in decreased HIF-1α translation (26). Under hypoxic conditions, HITT expression is repressed by HIF-1α–regulated MiR-205 expression, which is required for efficient HIF-1α mRNA translation and subsequent adaptive survival under hypoxia (26, 27). In contrast, in response to chemotherapeutic drug-induced double-strand breaks, HITT is transcriptionally activated and translocated to the nucleus, where it binds ataxia telangiectasia-mutated and attenuates the DNA damage-repair process by inhibiting the recruitment and activation of ataxia telangiectasia-mutated at sites of DNA breakage (28). These findings highlight the fact that lncRNA HITT can response to multiple stresses and that its subcellular localization dictates its specific biological functions.

Here, we report further analysis of the precise localization of HITT in the cytoplasm and the unexpected finding that HITT can localize to mitochondria. Our studies have revealed that HITT directly interacts with MFN2 and increases mitochondrial fragmentation by preventing MFN2 homotypic or heterotypic complex–mediated mitochondrial fusion, thus sensitizing cancer cells to UV-induced cell death.

## Results

### HITT regulates mitochondrial morphology

**Figure 1:**
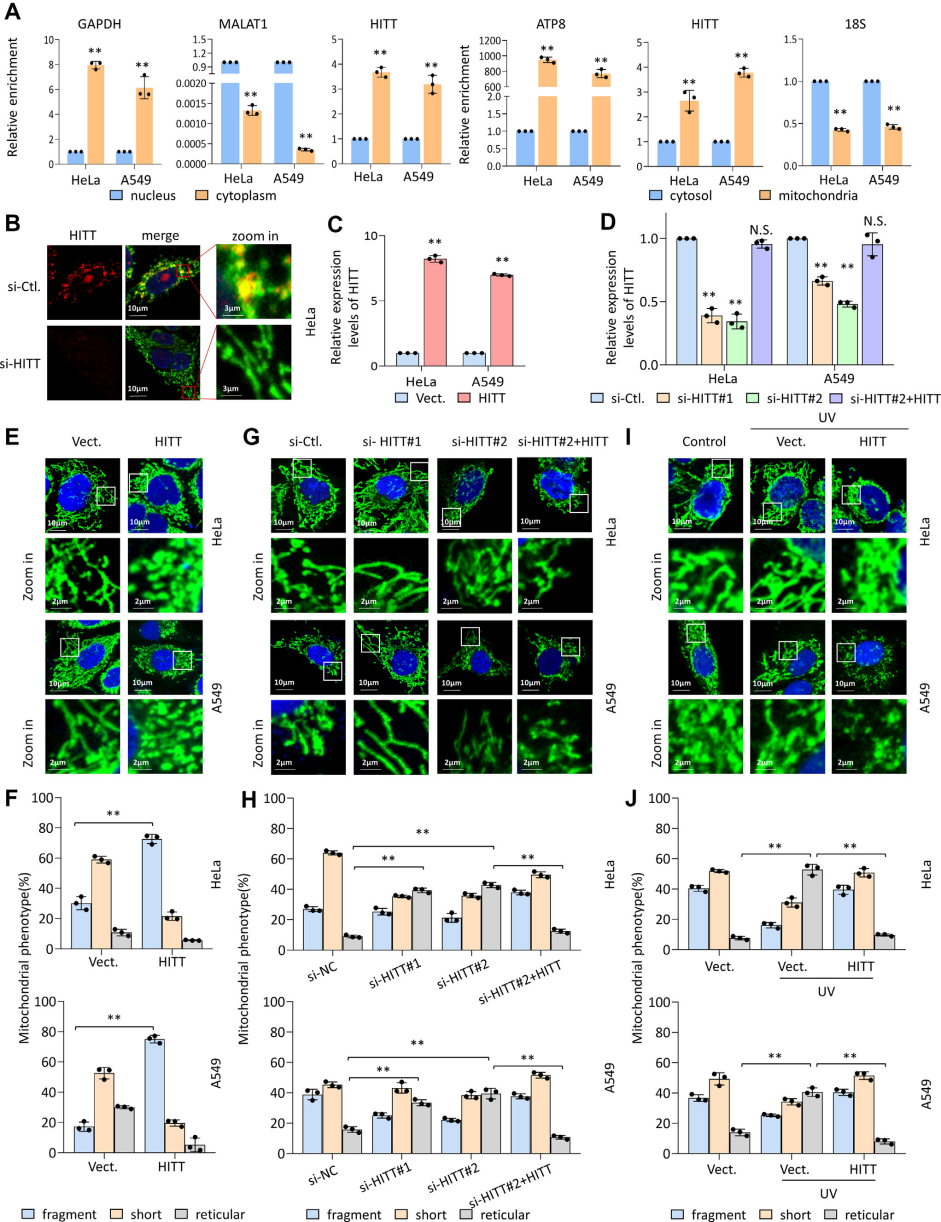
**HITT regulates mitochondrial morphology.***A*, the lncRNA HITT distribution in the cytoplasm and nucleus or cytosol and mitochondria were determined using qRT-PCR after fractionation. GAPDH, MALAT1, and ATP8 were used as cytoplasmic, nuclear, and mitochondrial markers, respectively. *B*, representative images of RNA-FISH staining of HITT and HSP60 in HeLa cells. *C* and *D*, HITT overexpression (*C*) or KD (*D*) efficiency was confirmed by qRT-PCR in HeLa and A549 cells, respectively. 18S was used as an internal control. *E*–*J*, representative confocal images of mitochondrial morphology as indicated by immunostaining with anti-HSP60 antibody (*green*) in HITT overexpression (*E* and *I*), KD or HITT rescued (*G*) HeLa and A549 cells with (*I*) or without (*E* and *G*) UV treatment. Quantification of mitochondrial morphology were presented in the bar graphs (*F*, *H*, and *J*). Scale bar in *E*, *G*, and *I* represents 10 μm. Data represented as means ± SEM in the bar graphs. ∗∗*p* < 0.01, N.S., not significant; (*A*, *C*, *D*, *F*, *H*, and J). See also Fig. S1. Vect., Vector. Ctl., control; HITT, HIF-1α inhibitor at translation level; HSP60, heat shock protein 60; KD, knockdown; qRT-PCR, quantitative real time-PCR.

HITT was distributed in both the nucleus and cytoplasm (Fig. 1*A*). Intriguingly, further fractionation and immunofluorescence revealed it was also localized in the mitochondria (Figs. 1, *A* and *B* and S1*A*). Nuclear, cytoplasmic, and mitochondrial markers (Histon3.1, α-tubulin, HSP60) were detected in Fig. S1*B*. This finding motivated us to investigate whether HITT influences mitochondrial morphology, which is vital to cell fate. To explore this, we first defined mitochondrial morphologies as fragmented, short, or reticular, as exemplified in Fig. S1*C*. The efficiencies of HITT overexpression or siRNA-mediated HITT knockdown (KD) were confirmed by quantitative real time-PCR (qRT-PCR) assay (Fig. 1, *C* and *D*). As indicated by heat shock protein 60 (HSP60) staining, mitochondria were distributed mainly at perinuclear regions in both HeLa and A549 cells, regardless of HITT levels (Fig. 1, *E*–*J*). Intriguingly, mitochondria were shortened with small ‘dotty’ morphology in HITT-overexpressing HeLa and A549 (Fig. 1, *E* and *F*), while the proportion of reticular mitochondria was significantly reduced (Fig. 1, *E* and *F*). This was validated by images captured by scanning electronic microscopy (Fig. S1*D*). In contrast, HITT KD by si-RNA or CRISPR/Cas9 resulted in connected, long tubular mitochondria in HeLa and A549 cell lines (Figs. 1, *G*, *H*, S1, *E* and *F*). Restoring HITT expression counteracted the altered mitochondrial morphology caused by HITT KD (Figs. 1, *G*, *H*, S1, *E* and *F*). Upon short-term mild exposure to stress, such as UV irradiation or treatment with the translational inhibitor cycloheximide or transcriptional inhibitor actinomycin-D, mitochondrial morphology is changed, mainly due to increased mitochondrial fusion, as suggested by previous reports (9). Under such conditions, HITT overexpression completely abolished the effects of such stresses on mitochondrial morphology (Figs. 1, *I*, *J* and S1, *G*–*J*). Therefore, HITT plays a role in regulating mitochondrial morphology under both basal and stressed conditions.

### HITT-regulated mitochondria dynamics are dependent on MFN2

**Figure 2:**
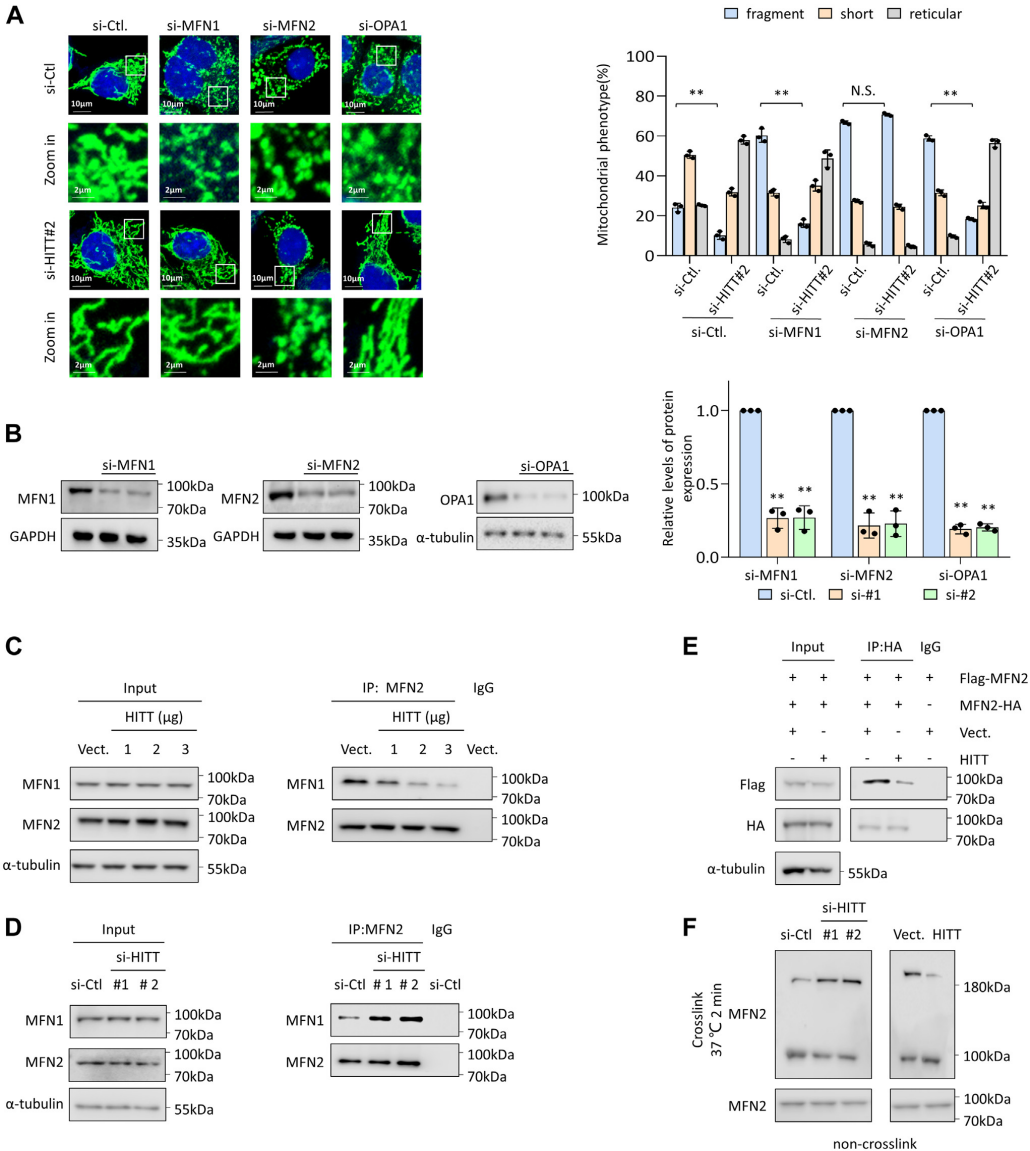
**HITT prevents MFN2 homotypic and heterotypic complex formation dependent on MFN2.***A* and *B*, representative confocal images of mitochondrial morphology as indicated by immunostaining with anti-HSP60 antibody (*green*) in the control and HITT KD HeLa cells with or without MFN1, MFN2, and OPA1 KD (*A*, *left*). Quantification of mitochondrial morphology were presented in the bar graphs (*A*, *right*).MFN1, MFN2, and OPA1 KD efficiencies were confirmed by Western blot assay (*B*, *left*). Quantification of Western blot was presented in the bar graphs (*B*, *right*). Scale Bar in A represents 10 μm. *C* and *D*, the interaction between MFN1 and MFN2 was determined by an immunoprecipitation (IP) assay in HITT dose overexpression (*C*) and KD (*D*) cells. IgG was used as a negative control. *E*, the MFN2 homotypic was analyzed by an IP assay Flag-MFN2 and Western blot analysis of MFN2-HA in Flag-MFN2 and MFN2-HA overexpression cells with or without HITT overexpression. IgG was used as a negative control. *F*, the relative levels of MFN2 dimers and monomers were analyzed by Western blot analysis following glutaraldehyde cross-linking in cell lysates generated from HITT-overexpressing or KD cells. Data represented as means ± SEM in the bar graphs. ∗∗*p* < 0.01; N.S., not significant. (*A* and *B*). See also Fig. S2. MFN, mitofusin; OPA, optic atrophy type; HITT, HIF-1α inhibitor at translation level; HSP60, heat shock protein 60; KD, knockdown.

We further asked how HITT regulates mitochondrial morphology. The mitochondria-shaping proteins, MFN1, MFN2, and OPA1, are required for mitochondria fusion. HITT did not affect the expression of MFN1, MFN2, and OPA1. As expected, KD of MFN1, MFN2, or OPA1 resulted in fragmented mitochondria (Fig. 2, *A* and *B*). Intriguingly, inhibition of HITT expression did not reverse MFN2 KD-induced mitochondrial fragmentation (Fig. 2, *A* and *B*). In contrast, HITT KD led to an elongated mitochondrial phenotype regardless of MFN1 KD or OPA1 KD. MFN2 overexpression resulted in reticular mitochondria. Intriguingly, MFN2 overexpression rescues HITT-induced mitochondrial fragmentation (Fig. S2*A*). After downregulating MFN1, MFN2, and OPA1, the expression of HITT remained unchanged (Fig. S2*B*). These data suggest that HITT acts mainly through interfering with the activity of MFN2.

### HITT prevents MFN2 homotypic and heterotypic complex formation

Next, we investigated how HITT influences MFN2 activity, which was unlikely to be by changing the levels of MFN2 expression (Fig. S2*C*). As homotypic or heterotypic complex formation is required for MFN2-mediated mitochondrial fusion, we reasoned that HITT may influence MFN2 homodimerization or its binding with MFN1. Intriguingly, an immunoprecipitation (IP) assay revealed that HITT overexpression reduced coprecipitation of MFN1 with anti-MFN2 precipitates from both HeLa and A549 cells (Fig. S2*D*). HITT also inhibits the binding of exogenous MFN1 and MFN2 (Fig. S2*E*). Dose-dependent inhibition was further revealed in transfected HeLa cells with increasing levels of HITT (Fig. 2*C*). In contrast, amounts of MFN1/MFN2 complex were elevated by siRNA-mediated HITT inhibition (Fig. 2*D*) and were also increased following 3 h UV treatment (Fig. S2*F*), which was in line with immunofluorescence results that showed that mitochondrial fusion was induced 3 h after UV exposure (Fig. S2*F*). Intriguingly, HITT overexpression suppressed UV-induced complex formation (Fig. S2*F*). Therefore, HITT inhibits the formation of MFN1/MFN2 heterotypic complexes.

The next question was whether HITT influences MFN2 homodimerization. To answer this, we generated two tagged MFN2 constructs, one with Flag and another with HA. Both constructs were simultaneously introduced into HeLa cells. Anti-HA IP assay revealed that levels of coprecipitated Flag-MFN2 were significantly lower in HITT-overexpressing cells than control cells (Fig. 2*E*). By contrast, HITT overexpression had little effect on MFN1 homotypic complexes (Fig. S2*G*). Furthermore, after cross-linking, two MFN2-containing complexes were detected under native conditions. Levels of MFN2 homodimers or heterodimers, a 200-kDa band, were much lower, while MFN2 monomers, a 100-kDa band, were higher in HITT-overexpressing HeLa cells than controls (Fig. 2*F*). The 200-kDa band was also detectable by the anti-MFN1 antibody (Fig. S2*H*), further suggesting that this 200-kDa band may represent both MFN2 homodimers and heterodimers. Thus, HITT inhibits MFN2 homodimerization or heterodimerization in cancer cells.

### HITT directly binds MFN2 at its dimerization domain

**Figure 3:**
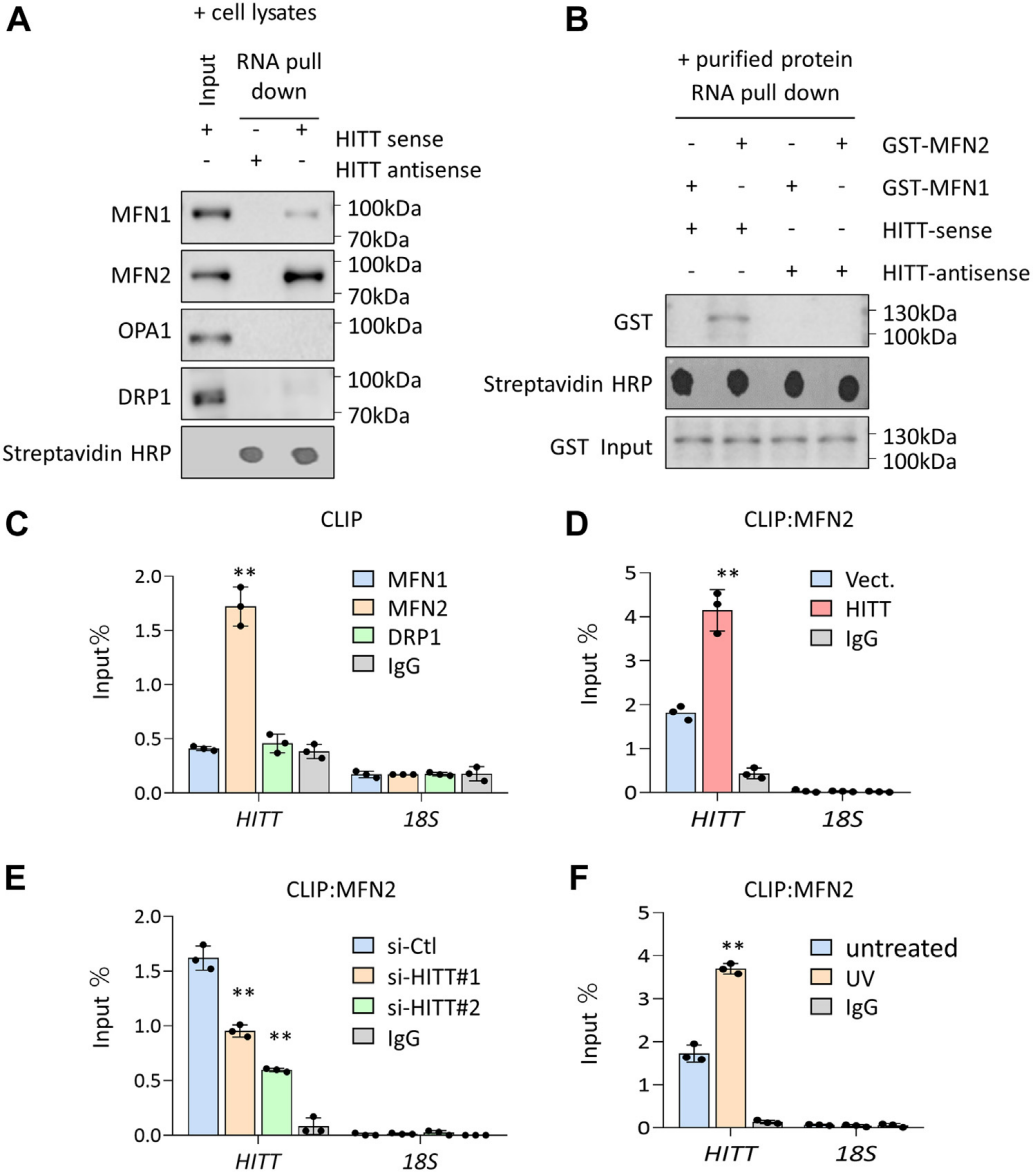
**HITT directly binds with MFN2.***A*, MFN1, MFN2, OPA1, and DRP1 levels in protein complexes immunoprecipitated by biotinylated HITT sense or antisense from whole-cell extracts of HeLa cells were determined by *in vitro* RNA pull-down assay combined with Western blot. The RNA pull-down efficiency was determined by dot blot showing the density of streptavidin HRP. *B*, the binding of biotinylated HITT with recombinant GST-MFN1 and GST-MFN2 were determined by biotinylated RNA pull-down assay *in vitro*. Biotinylated antisense HITT serves as a negative control. *C*, MFN1-, MFN2-, and DRP1-associated HITT was measured by CLIP in living HeLa cells. 18S mRNA and CLIP IgG were used as negative controls. *D*–*F*, MFN2-associated HITT was measured by CLIP MFN2 after HITT overexpression (*D*), KD (*E*), or UV treatment (*F*) in HeLa cells. 18S mRNA and CLIP IgG were used as negative controls. Data represented as means ± SEM in the bar graphs. ∗∗*p* < 0.01; compared with control. (*C*–*F*). CLIP, cross-linking and immunoprecipitation; DRP1, dynamin related protein 1; GST, glutathione S-transferase; HITT, HIF-1α inhibitor at translation level; KD, knockdown; MFN, mitofusin; OPA, optic atrophy type.

LncRNAs can exert their functions through RNA–protein interactions. To gain insight into the mechanisms underlying HITT-mediated MFN2 polymerization, we performed biotinylated RNA pull-down assays to identify potential HITT-binding proteins in HeLa cells. As shown, we found that sense HITT, but not an antisense control, specifically interacted with MFN1 and MFN2 (Fig. 3*A*). Neither sense nor antisense HITT bound OPA1 and dynamin-related protein 1. Direct interaction between MFN2/HITT was further validated by an in vitro–binding assay using *in vitro*–synthesized HITT and purified MFN2 and MFN1 proteins (Fig. 3*B*). Physical and specific interaction between MFN2 and HITT was also demonstrated in living cells *via* a UV cross-linking and immunoprecipitation assay (Fig. 3*C*). In addition, MFN2/HITT interaction was increased by HITT overexpression and decreased by HITT KD (Fig. 3, *D* and *E*). Furthermore, MFN2/HITT complex formation increased 3 h after UV exposure (Fig. 3*F*).

**Figure 4:**
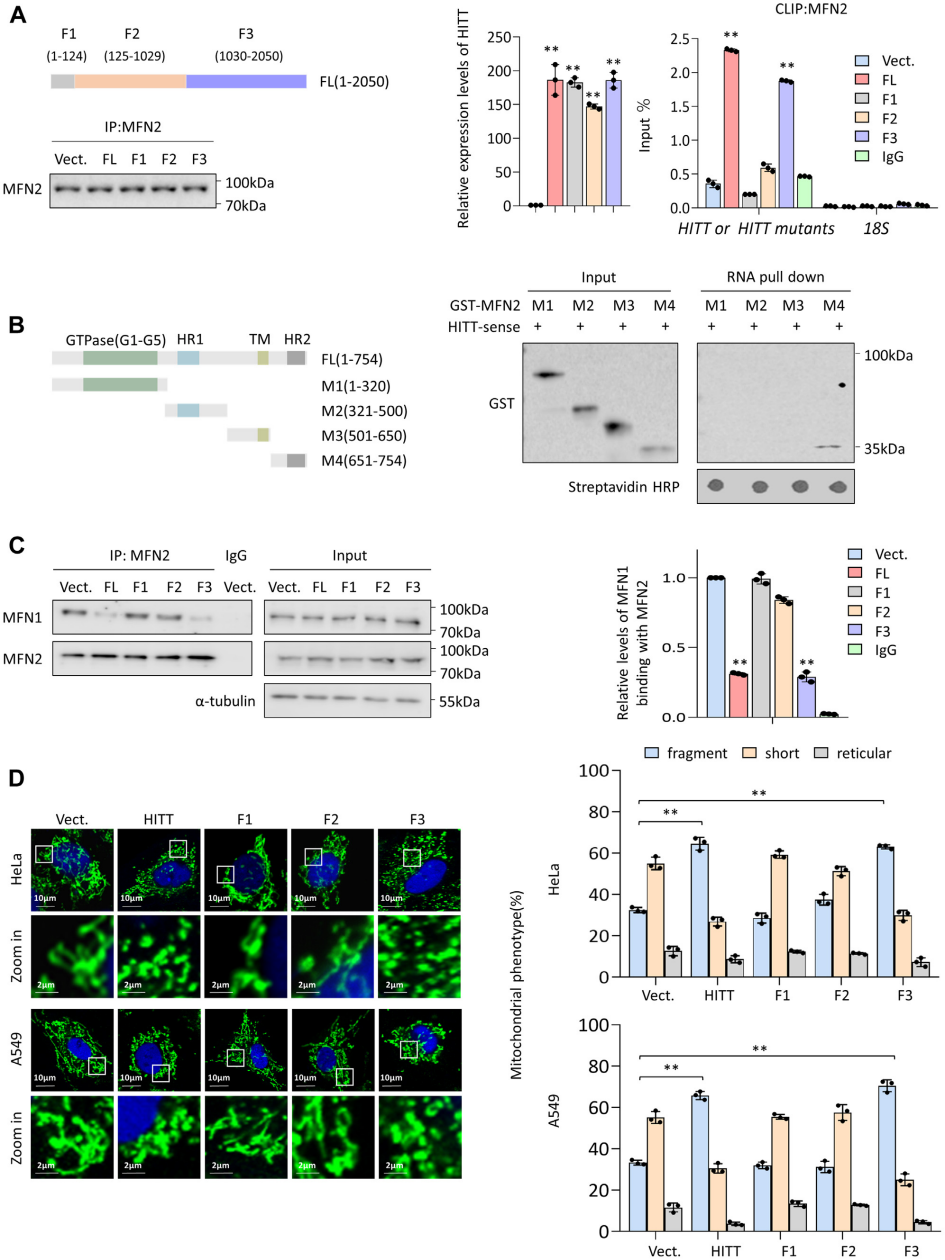
**HITT directly binds MFN2 at its dimerization domain.***A*, CLIP was used to detect the binding of MFN2 with full-length or HITT fragments, as indicated in the diagram (*left*, *top*). 18S mRNA and CLIP IgG were used as negative controls. MFN2 pull-down efficiency was determined by Western Blot (*left*, *bottom*). HITT fragment transfect efficiencies were measured by qRT-PCR (*middle*). *B*, the interaction between biotinylated HITT and GST-MFN2 or GST-MFN2 truncates, as indicated in the diagram (*left*), were determined by biotinylated RNA pull-down assay (*right*). The RNA pull-down efficiency was determined by dot blot showing the density of streptavidin HRP. *C*, the interaction between MFN1 and MFN2 was determined by an IP assay in full-length or HITT fragments overexpression cells. IgG was used as a negative control. Quantification of binding efficiency was presented in the bar graphs (*right*). *D*, representative confocal images of mitochondrial morphology as indicated by immunostaining with anti-HSP60 antibody (*green*) in full-length or HITT fragments overexpression cells (*left*). Quantification of mitochondrial morphology was presented in the bar graphs (*right*). Data represented as means ± SEM in the bar graphs. ∗∗*p* < 0.01. (*A*, *C*, and *D*). CLIP, cross-linking and immunoprecipitation; HITT, HIF-1α inhibitor at translation level; GST, glutathione S-transferase; HSP60, heat shock protein 60; IP, immunoprecipitation; MFN2, mitofusin-2; qRT-PCR, quantitative real time-PCR.

Given the physical and specific interaction between HITT/MFN2, we further explored the mechanisms underlying their interaction. To this end, HITT and fragmented HITT mutants were introduced into 4T1 cells, which do not endogenously express HITT. Cross-linking and immunoprecipitation assay of MFN2 showed that levels of MFN2’s binding with full-length HITT and Fragment 3 (F3; 1030 − 2050 nt) were similar, while binding to HITT fragments F1 and F2 was completely abolished (Fig. 4*A*). The key residues in MFN2 that mediated the binding of HITT were mapped to mutant 4 (M4) MFN2 (651 − 754 aa), a region-mediated oligomerization (29) (Fig. 4*B*). In line with our biochemical analysis, full-length and F3 HITT, but not F1 and F2, were able to localize at mitochondria and prevent complex formation between MFN1/MFN2 and mitochondrial fusion (Figs. S3, 4, *C* and *D*).

### HITT expression is suppressed by MiR-205 in stressed cancer cells

**Figure 5:**
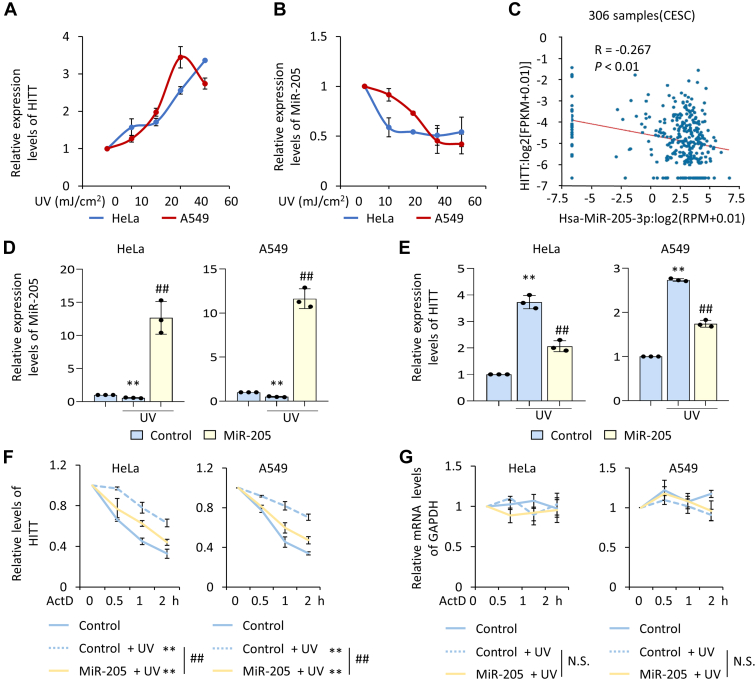
**HITT expression is suppressed by MiR-205 in stressed cancer cells.***A* and *B*, HITT (*A*) and MiR-205 (*B*) expression levels were measured by qRT-PCR after UV (0, 10, 20, 40, 60 mJ/cm2) treatment in HeLa and A549 cells. 18S or U6 was used as an internal control. *C*, correlation of HITT expression levels with MiR-205 expression in Cervical squamous cell carcinoma (CESC) by analyzing TCGA dataset in Starbase website (https://starbase.sysu.edu.cn/). *D* and *E*, relative expression levels of MiR-205 (*D*) and HITT (*E*) were determined by qRT-PCR after transfection of MiR-205 in HeLa and A549 cells with or without UV treatment. 18S or U6 was used as an internal control. *F* and *G*, the half-lives of HITT (*F*) and GAPDH (*G*) were determined by qRT-PCR after transfection of MiR-205 in HeLa and A549 cells with or without UV treatment in the presences of RNA synthesis inhibitor ActD. Data represented as means ± SEM in the bar graphs. ∗∗*p* < 0.01; compared with control. (*D*-*G*). ##, *p* < 0.01, relative to the UV treated control (*D* and *E*). See also Fig. S4. ActD, actinomycin D; HITT, HIF-1α inhibitor at translation level; qRT-PCR, quantitative real time-PCR; TCGA, The Cancer Genome Atlas.

Because HITT regulates the UV-induced hyperfusion, we asked whether HITT levels change with stress. Intriguingly, HITT levels were induced and MFN2 was not changed by UV irradiation in a dose-dependent manner (Figs. 5*A* and S4*A*). However, HITT promoter-driven luciferase reporter activity was unchanged with treatment (Fig. S4*B*), suggesting that the increased levels of HITT may be due to reduced RNA stability. miRNAs play essential roles in suppressing expression of mRNAs and lncRNAs. Therefore, we reasoned that HITT may be regulated by miRNA. Based on our previous data, four miRNAs (MiR-7, MiR-20, MiR-106, and MiR-205) were experimentally validated for inhibition of HITT expression following bioinformatic-prediction (26). However, MiR-205 was the only miRNA that had dramatically reduced expression following UV irradiation (Fig. S4*C*). Further dose-dependent analysis revealed that MiR-205 expression was gradually reduced with increasing doses of UV treatment (Fig. 5*B*), and this was inversely associated with increased HITT expression in UV-treated cells. Additionally, levels of HITT and MiR-205 were found to be negatively associated with each other in human cervical cancer tissues by analysis of The Cancer Genome Atlas database (Fig. 5*C*). Moreover, recovery of MiR-205 expression abolished UV-induced HITT expression (Fig. 5, *D* and *E*) and also accelerated the rate of HITT degradation (Fig. 5*F*). By contrast, in the same set of experiments, the control RNA, GAPDH mRNA, expression was unchanged with UV treatment or MiR-205 expression (Fig. 5*G*). MiR-205–overexpressing resulted in connected, long tubular mitochondria, whereas KD miR-205 was the opposite. Notably, neither overexpression nor KD MiR-205 affected the expression of MFN2 (Fig. S4, *D*–*F*). Overexpression of HITT abolished the effect MiR-205–induced mitochondrial fusion, while downregulation of MFN1/2 abrogated the effect of HITT/MiR-205 (Fig. S4*F*). Accordingly, overexpression of MiR-205 inhibited the formation of the MFN2-HITT complex and promoted the formation of the MFN1–MFN2 complex (Fig. S4, *G*–*H*). In addition, overexpression of HITT abolished the effect of MiR-205 on MFN1/MFN2 complex formation (Fig. S4*G*). These data collectively suggest that MiR-205 regulates MFN1/2/HITT complex formation by inhibiting HITT levels.

### The MiR-205/HITT/MFN2 axis regulates UV-induced apoptosis in cancer cells

It has been proposed previously that mitochondria elongation under stress is essential for the adaptive survival of cancer cells (12, 30). To investigate the role of the HITT in regulating adaptive survival to UV exposure *via* crystal violet staining assays, we constructed a model of adaptive survival to the death of UV-treated cells. We treated the cells with 40 mJ/cm2 UV and allowed the cells to recover for 0, 3, 6, 12, and 24 h to examine mitochondrial morphology and cell survival. We found that the mitochondrial morphology first became reticular, and then the mitochondria gradually fragmented over time (Fig. S5*A*). There was no obvious change in cell survival in the early stage, while the cell survival rate decreased in the late stage (Fig. S5*D*). Importantly, MFN2 expression was not changed in the early stage and slightly decreased in the late stage (Fig. S5*B*) and the expression of HITT was consistently elevated at the early stage and slightly decreased in the late stage (Fig. S5*C*), suggesting that HITT may be involved in this process. To demonstrate this, KD HITT was used to validate its effects on cell survival in this model. The results showed that downregulation of HITT prolonged the time of cell adaptive survival (Fig. S5*D*).

**Figure 6:**
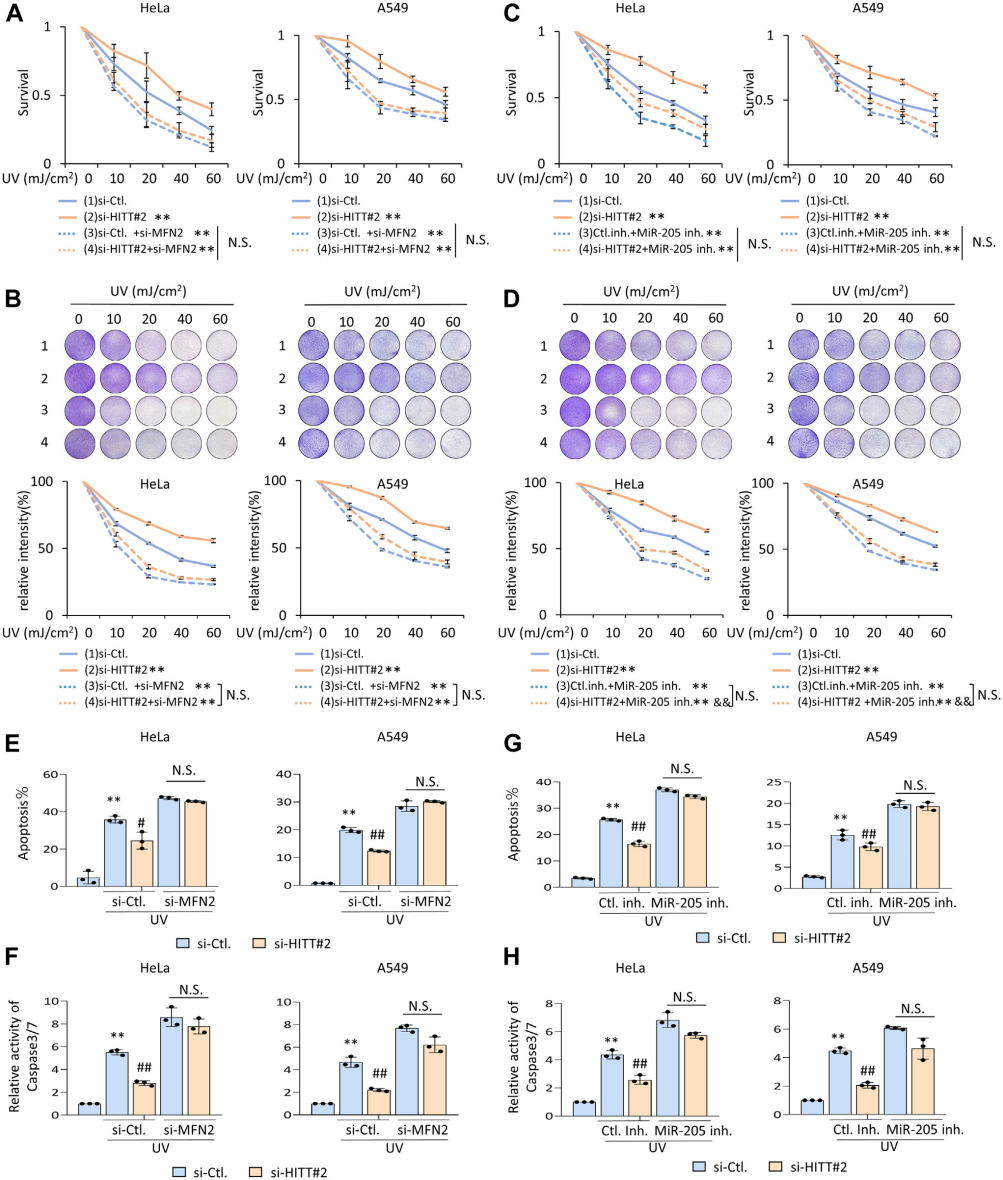
**The MiR-205/HITT/MFN2 axis regulates UV-induced apoptosis in cancer cells.***A*–*D*, cell survival rates were determined by MTT (*A* and *C*) and crystal violet staining (*B* and *D*) assays after KD MFN2 (A-B) or MiR-205 (*C* and *D*) in control or HITT KD cells under indicated treatment. *E*–*H*, cell death rates were determined by Annexin V staining (*E* and *G*) and caspase 3/7 activity (*F* and *H*) assays after KD MFN2 (*E* and *F*) or MiR-205 (*G* and *H*) in control or HITT KD cells under indicated treatment. Data represented as means ± SEM in the bar graphs. ∗∗*p* < 0.01; compared with si-Ctl. (*A*–*H*). & *p* < 0.01, relative to the si-HITT#2(*D*). #, *p* < 0.05. ##, *p* < 0.01, relative to the UV-treated si-Ctl(*E*–*H*). N.S., not significant (*A*–*H*). See also Fig. S5. HITT, HIF-1α inhibitor at translation level; KD, knockdown; MFN, mitofusin.

We investigated the role of the MiR-205/HITT/MFN2 axis in regulating survival to UV exposure *via* MTT (3-(4,5)-dimethylthiahiazo (-z-y1)-3,5-di-phhenytetrazoliumromide) and crystal violet staining assays. HITT KD decreased UV-induced cell death and the effect was largely diminished by MFN2 KD in both HeLa and A549 cells (Fig. 6, *A* and *B*). Inhibition of MiR-205, an upstream regulator of HITT, led to increased HITT expression and increased the number of cells that underwent UV-induced cell death (Fig. 6, *C* and *D*). HITT expression abolished the anti-cell death effect of HITT KD under the UV treatment (Fig. S5*E*). These data collectively suggest that the MiR-205/HITT/MFN2 axis plays essential roles in regulating UV-induced cell death.

We also found that MiR-205/HITT/MFN2 axis-regulated cell death results from increased apoptotic sensitivity. Annexin V staining and cleaved caspase 3/7 assays showed that the apoptotic rates of HeLa and A549 cells were increased following UV treatment and were decreased by HITT KD. HITT-promoted and UV-induced apoptosis was compromised by MFN2 KD and strengthened by MiR-205 inhibition (Fig. 6, *E*–*H*). Therefore, the MiR-205/HITT/MFN2 axis plays essential roles in regulating UV-induced apoptosis.

## Discussion

The discovery and characterization of lncRNAs has increased our understanding of diverse cellular processes (31). However, whether lncRNAs affect mitochondria fusion remains poorly understood. Here, we report lncRNA HITT specifically interacts with the mitochondrial outer membrane protein MFN2 and regulates mitochondrial fusion by disrupting MFN2 interaction with itself or MFN1. In addition, we suggest a model for how lncRNA HITT regulates mitochondrial dynamics in response to stress and confers sensitivity to stress-induced apoptosis.

The connection between lncRNAs and mitochondrial dynamics has been suggested in previous studies (32, 33). The lncRNA Malat1 has been shown to promote fusion *via* the MiR-26b/MFN1 axis (34), while lncRNA plscr4 acts *via* MiR-214/MFN2 (35). However, to our knowledge, HITT is the first mitochondrial lncRNA to date to be identified as a regulator of mitochondrial fusion. Organelle-specific lncRNAs have gained a lot of attention in recent years (23). Like proteins, the functions of lncRNAs are closely correlated with their subcellular localizations, because their functions are directed by local molecular interactions (36). For example, lncRNAs modulate transcriptional programs through chromatin interactions in the nucleus and mediate signal transduction pathways, translational programs, and posttranscriptional control of gene expression in the cytoplasm (18). In addition, mitochondria-localized lncRNAs have been shown to regulate mitochondrial functions (23). For example, the lncRNA RMRP, once bound to HuR, is exported out of the nucleus and targeted to the mitochondria *vi*a interaction with the mitochondrial protein growth arrest-specific protein 1 (GRSF1) (37). As such, it regulates oxygen consumption rates and mitochondrial DNA replication. Mitochondrial fractionation has demonstrated that lncRNA GAS5 (growth arrest-specific transcript 5) localizes to the mitochondria, where it specifically binds malate dehydrogenase 2, a rate-limiting enzyme in the mitochondria, and regulates the rate of metabolism *via* the tricarboxylic acid cycle (23). We demonstrate here that HITT is another mitochondria-localized lncRNA that regulates mitochondrial dynamics. Although lncRNAs display sequence and/or positional conservation between human and mouse embryonic stem cells, they can be processed differently, which results in their localization to different subcellular compartments and enabling them to have distinctive functions (38). Our study emphasizes the role of cellular localization in the functions of lncRNAs. Although the ability of HITT to regulate mitochondrial fusion is controlled by MiR-205 upon UV treatment, HITT’s ability to bind MFN2 may enable its ability to localize to mitochondria and, thus, its regulating of mitochondrial fusion. There is no mitochondria-specific RNA sequence identified so far, whether other RNA-binding proteins facilitate the translocation of HITT from either the nucleus or cytoplasm to the mitochondria warrants further investigation. It should be noted that HITT is also localized in the nucleus. Although the ability of HITT in regulating mitochondria morphology is mainly dependent on MFN2, whether nuclear HITT is involved in regulating the process is worth investigating in the future. Such mechanisms may not only provide an additional layer of regulation for HITT/MFN2-controlled fusion but also provide insight into how cytoplasmic or nuclear signals are transduced to the mitochondria.

It is known that MFN2 can mediate ER−mitochondrial connection, thereby increasing the efficiency of mitochondrial calcium uptake. The mechanism underlying ER−mitochondrial connection is similar to that of mitochondria fusion and is mediated by MFN2 dimerization (39). As such, it is possible that HITT may assist in the regulation of mitochondria-associated membrane formation and related functions, such as the maintenance of calcium homeostasis.

Although a role of MiR-205 in human cancer is controversial (40, 41), overexpression of MiR-205 in human corneal epithelial cells has been shown to protect against UV-induced corneal damage by targeting Toll-like receptor 4 (TLR4) (42). Our data indicate that HITT may be another downstream effector of MiR-205–regulated cell death in response to UV irradiation. MFN2 acts as an antiapoptotic effector in cells exposed to UV radiation. Consistent with our data, cells in which MFN2 is overexpressed have been shown to tolerate apoptosis, and inhibition of MFN2-mediated fusion renders cell more responsive to apoptotic stimuli (43, 44). Under stress conditions, inhibition of MiR-205–regulated HITT degradation may function as a switch to shift the balance of mitochondrial dynamics from mitochondrial fusion to fission and potentiate apoptosis in an MFN2-dependent manner if the stress is unresolved. It should be also noted that multiple functions of MFN2 have been identified, including the regulation of cancer metastasis (45) and the immune response (46). HITT directly interacts with MFN2, so its involvement in the different functions of MFN2 needs further exploration.

In summary, we have identified a mechanism of lncRNA-regulated mitochondria fusion. When localized to mitochondria, the lncRNA HITT specifically interacts with a key mediator of mitochondrial fusion, MFN2, and inhibits its ability to form homotypic and heterotypic complexes. Under UV-induced stress, HITT stabilization tips the balance of mitochondrial dynamics toward fission, which sensitizes the cells to apoptosis (Fig. S6).

## Experimental procedures

### Cell culture and treatments

The human cervical cancer cell HeLa, mouse breast cancer cell 4T1, and human lung cancer A549 cells were bought from ATCC and cultured in Dulbecco’s modified Eagle’s medium supplemented with 10% (v/v) fetal bovine serum (Biological industries). All cell lines were grown at 37 °C in the humidified incubator with 5% CO2. All cell lines were authenticated and characterized by the supplier and were monitored regularly for their authenticity by Genetic Testing Biotechnology Corporation and to be free of *mycoplasma* contamination. Cycloheximide was used in 10 μM and actinomycin-D was used in 5 μM for 2 h.

### Nuclear and cytoplasm fractionation

Cytoplasm lysis buffer (10 mM Hepes, pH7.9, 10 mM KCl, 1.5 mM MgCl2, 0.5 mM β-mercaptoethanol) was added to HeLa and A549 cells, followed by moderate vortex for 15 s (s) and 15 to 20 min (min) incubation on ice. 0.1 % Triton X 100 was then added to the mixture followed by another round of vortex and incubation. The cytoplasm fraction was obtained by collecting supernatant after centrifugation at 16, 000*g* for 10 min. The resulting pellet was lysed in the nuclear fraction buffer (10 mM Hepes, pH7.6, 1 mM DTT, 7.5 mM MgCl2, 0.2 mM EDTA, 0.3 mM NaCl, 1 M Urea, 1% NP-40). The supernatant was collected as the nuclear fraction by centrifugation at 16, 000*g* for 30 min.

### Subcellular organelle fractionation

Cells were collected and lysed in MTE buffer (270 mM D-mannitol, 10 mM Tris–HCl, pH 7.4, 0.1 mM EDTA) with 1 mM PMSF. After ultrasonic crushing, the lysate was centrifugated at 1, 400*g* for 10 min to yield nucleus-enriched pallet. The supernatant was collected and centrifuged at 15, 000*g*, 4 °C for 20 min to separate crude ER supernatant from crude mitochondria (pellet). The supernatant was then transferred to the ultracentrifugation tube which pre-added sucrose concentration gradient and centrifuged at 1, 52, 000*g*, 4 °C for 70 min. Through ultracentrifugation, the top part of solution was collected as the cytosol and the median portion–observed ‘white band’ was collected by syringe and centrifuged at 1, 26, 000*g* for 45 min. The pellet was obtained as ER fraction. The previously mentioned coarse mitochondria were resuspended with MTE, and the supernatant was then loaded on a sucrose gradient and centrifuged at 1, 52, 000*g* for 70 min. The portion of mitochondrial band was collected and centrifuged at 15, 000*g* to get yellow precipitates. The RNA was purified from the fractionated precipitate by adding Trizol and the resulting RNA was analyzed by RT-PCR.

### RNA-fluorescence in situ hybridization

Cells grown on cover slips in a 24-well plate were fixed in 4% paraformaldehyde (PFA) solution for 20 min after three washes in 1 × PBS. Cells were permeabilized with 0.1% Triton X-100 solution on ice for 4 min. Next, the microarray was prehybridized in hybridization buffer (50% deionized formamide, 5 × SSC, 5 × Denhardt’s, 250 μg/ml yeast tRNA, 500 μg/ml sperm DNA) at 37 °C for 1 h. After that, cells were incubated with 200 to 300 ng/ml biotin-labeled anti-lncRNA HITT oligodeoxynucleotide probe at 37 °C for 3 h. The microarray was then washed three times for 5 min each in 5 × SSC, 2 × SSC, and 0.2 × SSC at 37 °C. After blocking in blocking buffer (10% milk dissolved in maleic acid buffer), the microarray was incubated in Streptavidin Alexa Fluor-555 secondary antibody at room temperature for 1 h. DAPI (4′,6-diamidino-2-phenylindole) is used as a nuclear counterstain. The ﬂuorescence was measured by a laser scanning confocal microscope (Zeiss LSM880 With Airyscan).

### Construction of the expression plasmid

The coding sequence of MFN1 and MFN2 genes with different homologous arms were amplified by PCR using DNA templates derived from HEK293 cells. The PCR products were inserted into pcDNA3.1 vector to generate pcDNA3.1-Flag-MFN1, pcDNA3.1-Flag-MFN2, pcDNA3.1-MFN1-HA, pcDNA3.1-MFN2-HA plasmid constructs. The coding regions were subcloned to pGEX-6P-1 glutathione S-transferase (GST) expression vector and the constructs transformed into *E. coli* BL21 to generate GST fusion proteins.

### siRNA and CRISPR/Cas9 plasmid transfection

si-RNA and PX458 plasmids were transfected into cells by Lipofectamine 2000 (Invitrogen) following the manufacturer’s instructions. We used a modified version of the CRISPR/Cas9 plasmid pSpCas9(BB)-2A-GFP (PX458) that was engineered to contain a puromycin expression cassette and BbsI and BsaI cloning sites for insertion of two double-stranded oligonucleotides respectively encoding two different sgRNAs, which specifically target two distinctive sites in HITT gene. The sgRNAs sequences were obtained from UCSC database (http://genome.ucsc.edu/). The paired oligonucleotides sequence (sgRNA) specifically targeting either HITT full-length or HITT exon 3 are shown in Table S1. CRISPR/Cas9-mediated HITT KD mixture colon were collected after adding puromycin. si-RNA specifically targeting HITT, MFN1, MFN2, OPA1, and nonspecific si-scramble control were synthesized by GenePharma. The siRNA oligos and mimics sequences are listed as follows: si-Ctl (UUUUCCGAACGUGUCACGUTT); si-HITT#1(CCAGGAAGGCGAUUUACAATT); si-HITT#2 (CCUCAUGAAUGGGAUUAAUTT); si-MFN1 (GGAUCACAUUUUGUUGAAGTT); si-MFN2 (ACACAUGGCUGAAGUGAAU); si-OPA1 (GUUAUCAGUCUGAGCCAGGTT).

### Immunofluorescence staining

After adhering to cover slips in a 24-well plate, cells were treated with 4% PFA to fix morphology for 15 min. Fixed cells were treated with 0.1% Triton X-100 solution for 5 min. Then cells were blocked by 3% bovine serum albumin for 1 h followed by the anti-HSP60 antibody incubation at 4 °C overnight. Cells were washed in 1× PBS and then incubated them with the fluorescently labeled secondary antibody for 1 h in the dark. DAPI (4′,6-diamidino-2-phenylindole) was stained for 3 min for visualizing the nucleus. The slices were mounted by 90% glycerin. Images were captured by Zeiss Axio Observe confocal microscope. Typically, the antibody of HSP60 was used at 1:100 and the secondary antibody was used at 1:400 dilutions in blocking solution.

### Mitochondria morphology analysis

The mitochondria images were analyzed by Image J. Briefly, the mitochondrial morphology was defined as described previously (9). Briefly, fragment mitochondria were defined as small, spherical, and isolated mitochondrial with visible free ends. Reticular mitochondria were defined as connected, long tubular mitochondria, the ends of which are difficult to visualize. Short mitochondria were defined as the mitochondria between fragment and reticular mitochondria. At least 10 cells for each treatment were analyzed and quantified.

### Western blot

Cells were lysed in UREA buffer (8 M Urea, 1 M Thiourea, 0.5% CHAPS, 50 mM DTT, and 24 mM Spermine). Same amount proteins were separated by SDS PAGE. After being incubated with the indicated primary and secondary antibodies, the immune complex signals were detected by ECL kit. Antibodies used for Western blot were listed as follows: MFN1 (Abclonal, A9880), MFN2 (Abclonal, A12771), OPA1 (Santa Cruz Biotechnology, sc-393296), dynamin-related protein 1 (Santa Cruz Biotechnology, sc-101270), GAPDH (Proteintech, Cat.#60004-1-Ig), Flag (Proteintech, 20543-1-AP), α-tubulin (Proteintech, 66031-1-Ig), GST (Abclonal, AE006).

### RNA extraction and qRT-PCR

Total RNA was extracted from cells using Trizol Reagent following the manufacturer’s instructions (Invitrogen). PrimeScript reverse transcription reagent kit (TaKaRa, #RR047A) was used in the presence of gDNA Eriser to synthesize cDNA. qRT-PCR was performed in the ViiA7 real-time PCR (Applied Biosystems) using SYBR Premix Ex Taq II kit for Real-Time (TaKaRa). HITT are divided into three fragments (F1, F2, F3) according to HITT exons. The primer sequences used in qRT-PCR are listed in Table S1.

### Immunoprecipitation

Cells were lysed in NETN buffer (50 mM Tris–HCl [pH 8.0], 150 mM NaCl, 1% NP-40, 1 mM EDTA), with Proteinase Inhibitor Cocktail (MedChemExpress, #HY-K0010) added freshly before use. Then precleaned protein G sepharose beads (GE Health care) were added to the resulting lysate at 4 °C. After being precleaned, specific antibodies or control IgG was added to the supernatant, which was incubated with fetal bovine serum–blocked beads for at least 20 h at 4 °C. Beads with the bound immunoprecipitates were collected and subjected to four time washes with the cold NETN. The final immunoprecipitates were extracted for Western Blot assay.

### *In vitro* RNA pull-down assay

Biotin-labeled HITT and its antisense were *in vitro* synthesized by Biotin RNA Labeling Mix (Roche). After treatment with RNase-free DNase I, Biotin-Labeled RNA was heated at 70 °C for 15 min and then cooled down on ice for 2 min. The secondary structure recovered Biotin-labeled RNA incubated with streptavidin agarose beads (Invitrogen) at 4 °C for 2 h. RNA-captured beads were incubated with the fresh cell lysates or GST-fused proteins at 4 °C for 1 h. Then the beads were boiled in SDS-loading buffer and detected by Western Blot.

### UV-cross-linking RNA-IP

Cells were washed before UV-cross-linked at 400 mJ/cm2 and collected in lysis buffer (5 mM Pipes [PH 8.0], 85 mM KCl, 0.5% NP40 and 1% SDS, 10 mM EDTA, 50 mM Tris–HCl [PH 8.1]) and supplemented with Protease Inhibitor Cocktail and RNase inhibitor (Thermo Fisher Scientific). After precleared with protein A/G sepharose beads, cell lysates were treated with the indicated antibodies or IgG control at 4 °C overnight. Then, the antibody–RNA complexes were collected by using the blocked Protein A/G sepharose beads. The immunoprecipitated RNA was eluted and isolated for the subsequent reaction to reverse transcribed into first-strand cDNA that will be used for further qRT-PCR analysis for the target RNA of interest.

### Luciferase reporter assay

The cells were transfected with a plasmid mixture including Renilla luciferase and pGL3-HITT-promoter followed by UV treatments. The luciferase activities were detected with the Dual Luciferase Reporter Assay System (Promega, #E1910), according to the manufacture’s instruction. The relative luciferase activities were normalized with the Renilla luciferase activities.

### MTT assay

After transfection and UV treatment, cell viabilities were assessed by the colorimetric MTT (Sigma-Aldrich) assay. The final concentration of cells were incubated with MTT working solution at 0.5 mg/ml at 37 °C for 4 h. Formazan was dissolved with 100 μl dimethyl sulfoxide (Sigma-Aldrich) after removing the supernatant. The absorbance was measured with a spectrometer at 490 nm. Each experiment was conducted in triplicates and repeated independently for three times.

### Crystal violet assay

The cells were fixed by 4% PFA for 20 min, washed with 1× PBS followed by 20 min incubation with 1% crystal violet. After several rounds of washes in 1× PBS, cells were visible clearly in deep purple. Quantitative analyses of intensity were used by Image J software in three independent experiments.

### Apoptosis assay

After transfection, cells were treated with different intensities of UV and restored for 18 h, both suspension and attached cells were collected. Cell suspension in binding buffer was stained with Annexin V/FITC for 10 min and propidium iodide for 5 min at room temperature. The rate of apoptosis was analyzed by flow cytometry within 1 h after staining.

### Caspase3/7 activity assay

Following the indicated treatments, cells were subjected to the caspase 3/7 activity assay by Caspase-Glo 3/7 Assay Systems (Promega, #G8091) according to the manufacturer’s instructions. Three independent experiments were conducted, which were represented as a fold increase of fluorescence calculated by comparing cells with untreated control cells.

### Transmission electron microscopy

Cells were dissected and fixed with 2% PFA and 0.2% glutaraldehyde in sodium cacodylate buffer (pH 7.4) at 37 °C for 1 h, and then dehydrated in a graded ethanol series and embedded in epoxy resin. Ultrathin sections (60–80 nm) were mounted on nickel grids. The samples were observed with a transmission electron microscope (Tecnai G2).

### Statistical analysis

Statistical analysis was done by GraphPad software, version 7 (https://www.graphpad.com/). Data are presented as the means ± SEM. Student *t* test was applied to assess the statistical significance between two groups and Chi-square test was used to assess the statistical significance among three or more groups. *p* values <0.05 were considered significant.

## Data availability

The authors confirm that the data supporting the findings of this study are available within the article and its supporting information.

## Supporting information

This article contains supporting information.

## Conflict of interests

The authors declare that they have no competing interests.

## References

[1] Nunnari J., Suomalainen A. (2012). Mitochondria: in sickness and in health. Cell.

[2] Liu Y., Shi Y. (2020). Mitochondria as a target in cancer treatment. MedComm.

[3] Kraus F., Roy K., Pucadyil T.J. (2021). Function and regulation of the divisome for mitochondrial fission. Nature.

[4] Dorn G.W. (2019). Evolving concepts of mitochondrial dynamics. Annu. Rev. Physiol..

[5] Gao S., Hu J. (2021). Mitochondrial fusion: the machineries in and out. Trends Cell Biol..

[6] Züchner S., Mersiyanova I.V., Muglia M., Bissar-Tadmouri N., Rochelle J., Dadali E.L. (2004). Mutations in the mitochondrial GTPase mitofusin 2 cause Charcot-Marie-Tooth neuropathy type 2A. Nat. Genet..

[7] Stuppia G., Rizzo F., Riboldi G., Del Bo R., Nizzardo M., Simone C. (2015). MFN2-related neuropathies: clinical features, molecular pathogenesis and therapeutic perspectives. J. Neurol. Sci..

[8] Eisner V., Picard M. (2018). Mitochondrial dynamics in adaptive and maladaptive cellular stress responses. Nat. Cell Biol..

[9] Tondera D., Grandemange S., Jourdain A., Karbowski M., Mattenberger Y., Herzig S. (2009). SLP-2 is required for stress-induced mitochondrial hyperfusion. EMBO J..

[10] Youle R.J., van der Bliek A.M. (2012). Mitochondrial fission, fusion, and stress. Science (New York, N.Y.).

[11] Perfettini J.L., Roumier T., Kroemer G. (2005). Mitochondrial fusion and fission in the control of apoptosis. Trends Cell Biol..

[12] Gomes L.C., Di Benedetto G., Scorrano L. (2011). During autophagy mitochondria elongate, are spared from degradation and sustain cell viability. Nat. Cell Biol..

[13] Gomes L.C., Scorrano L. (2011). Mitochondrial elongation during autophagy: a stereotypical response to survive in difficult times. Autophagy.

[14] Rambold A.S., Kostelecky B., Elia N., Lippincott-Schwartz J. (2011). Tubular network formation protects mitochondria from autophagosomal degradation during nutrient starvation. Proc. Natl. Acad. Sci. U. S. A..

[15] Gomes L.C., Di Benedetto G., Scorrano L. (2011). Essential amino acids and glutamine regulate induction of mitochondrial elongation during autophagy. Cell Cycle (Georgetown, Tex.).

[16] Kopp F., Mendell J.T. (2018). Functional classification and experimental dissection of long noncoding RNAs. Cell.

[17] Palazzo A.F., Koonin E.V. (2020). Functional long non-coding RNAs evolve from junk transcripts. Cell.

[18] Yao R.W., Wang Y., Chen L.L. (2019). Cell functions long noncoding RNAs. Nat. Cell Biol..

[19] Statello L., Guo C.J., Chen L.L. (2021). Gene regulation by long non-coding RNAs and its biological functions. Nat. Rev. Mol. Cell Biol..

[20] Ransohoff J.D., Wei Y., Khavari P.A. (2018). The functions and unique features of long intergenic non-coding RNA. Nat. Rev. Mol. Cell Biol..

[21] Anastasiadou E., Jacob L.S., Slack F.J. (2018). Non-coding RNA networks in cancer. Nat. Rev. Cancer.

[22] Slack F.J., Chinnaiyan A.M. (2019). The role of non-coding RNAs in oncology. Cell.

[23] Sang L., Ju H.Q. (2021). Mitochondrial long non-coding RNA GAS5 tunes TCA metabolism in response to nutrient stress. Nat. Metab..

[24] Fazal F.M., Han S., Parker K.R., Kaewsapsak P., Xu J., Boettiger A.N. (2019). Atlas of subcellular RNA localization revealed by APEX-seq. Cell.

[25] Yamazaki T., Souquere S., Chujo T., Kobelke S., Chong Y.S., Fox A.H. (2018). Functional domains of NEAT1 architectural lncRNA induce paraspeckle assembly through phase separation. Mol. Cell.

[26] Wang X., Li L., Zhao K., Lin Q., Li H., Xue X. (2020). A novel LncRNA HITT forms a regulatory loop with HIF-1α to modulate angiogenesis and tumor growth. Cell Death Differ..

[27] Wang X., Wang Y., Li L., Xue X., Xie H., Shi H. (2020). A lncRNA coordinates with Ezh2 to inhibit HIF-1α transcription and suppress cancer cell adaption to hypoxia. Oncogene.

[28] Zhao K., Wang X., Xue X., Li L., Hu Y. (2020). A long noncoding RNA sensitizes genotoxic treatment by attenuating ATM activation and homologous recombination repair in cancers. PLoS Biol..

[29] Koshiba T., Detmer S.A., Kaiser J.T., Chen H., McCaffery J.M., Chan D.C. (2004). Structural basis of mitochondrial tethering by mitofusin complexes. Science (New York, N.Y.).

[30] Li J., Huang Q., Long X., Guo X., Sun X., Jin X. (2017). Mitochondrial elongation-mediated glucose metabolism reprogramming is essential for tumour cell survival during energy stress. Oncogene.

[31] Batista P.J., Chang H.Y. (2013). Long noncoding RNAs: Cellular address codes in development and disease. Cell.

[32] Tian T., Lv X., Pan G., Lu Y., Chen W., He W. (2019). Long noncoding RNA MPRL promotes mitochondrial fission and cisplatin chemosensitivity *via* disruption of pre-miRNA processing. Clin. Cancer Res..

[33] Wang K., Long B., Zhou L.Y., Liu F., Zhou Q.Y., Liu C.Y. (2014). CARL lncRNA inhibits anoxia-induced mitochondrial fission and apoptosis in cardiomyocytes by impairing miR-539-dependent PHB2 downregulation. Nat. Commun..

[34] Chen Y., Li S., Zhang Y., Wang M., Li X., Liu S. (2021). The lncRNA Malat1 regulates microvascular function after myocardial infarction in mice *via* miR-26b-5p/Mfn1 axis-mediated mitochondrial dynamics. Redox Biol..

[35] Lv L., Li T., Li X., Xu C., Liu Q., Jiang H. (2018). The lncRNA Plscr4 controls cardiac hypertrophy by regulating miR-214. Mol. Ther. Nucl. Acids.

[36] Bridges M.C., Daulagala A.C., Kourtidis A. (2021). LNCcation: lncRNA localization and function. J. Cell Biol..

[37] Noh J.H., Kim K.M., Abdelmohsen K., Yoon J.H., Panda A.C., Munk R. (2016). HuR and GRSF1 modulate the nuclear export and mitochondrial localization of the lncRNA RMRP. Genes Dev..

[38] Chen L.L. (2016). Linking long noncoding RNA localization and function. Trends Biochem. Sci..

[39] de Brito O.M., Scorrano L. (2008). Mitofusin 2 tethers endoplasmic reticulum to mitochondria. Nature.

[40] Qin A.Y., Zhang X.W., Liu L., Yu J.P., Li H., Wang S.Z. (2013). MiR-205 in cancer: an angel or a devil?. Eur. J. Cell Biol..

[41] Wang X., Yu M., Zhao K., He M., Ge W., Sun Y. (2016). Upregulation of MiR-205 under hypoxia promotes epithelial-mesenchymal transition by targeting ASPP2. Cell Death Dis..

[42] Fu J.Y., Yu X.F., Wang H.Q., Lan J.W., Shao W.Q., Huo Y.N. (2020). MiR-205-3p protects human corneal epithelial cells from ultraviolet damage by inhibiting autophagy *via* targeting TLR4/NF-κB signaling. Eur. Rev. Med. Pharmacol. Sci..

[43] Sugioka R., Shimizu S., Tsujimoto Y. (2004). Fzo1, a protein involved in mitochondrial fusion, inhibits apoptosis. J. Biol. Chem..

[44] Neuspiel M., Zunino R., Gangaraju S., Rippstein P., McBride H. (2005). Activated mitofusin 2 signals mitochondrial fusion, interferes with Bax activation, and reduces susceptibility to radical induced depolarization. J. Biol. Chem..

[45] You M.H., Jeon M.J., Kim S.R., Lee W.K., Cheng S.Y., Jang G. (2021). Mitofusin-2 modulates the epithelial to mesenchymal transition in thyroid cancer progression. Sci. Rep..

[46] Lloberas J., Muñoz J.P., Hernández-Álvarez M.I., Cardona P.J., Zorzano A. (2020). Macrophage mitochondrial MFN2 (mitofusin 2) links immune stress and immune response through reactive oxygen species (ROS) production. Autophagy.

