# Supplementary material for: Mitochondria-localized lncRNA HITT inhibits fusion by attenuating formation of mitofusin-2 homotypic or heterotypic complexes
Source: J Biol Chem. 2022 Dec 23;299(2):102825. doi: 10.1016/j.jbc.2022.102825 (PMC9867983; doi:10.1016/j.jbc.2022.102825)
Supplement: Supporting information [file mmc1.pdf]

Mitochondria-localized lncRNA HITT inhibits fusion by attenuating MFN2 homo- or heterotypic complexes

Xingwen Wang[1,#], Yi Zhang[1,#], Qingyu Lin[1], Kunming Zhao[1], Ying Hu[1,*]

[1]School of Life Science and Technology, Harbin Institute of Technology, Harbin, Heilongjiang Province, China, 150001.

[2]* To whom correspondence should be addressed. Tel: 0086-86403826; Fax: +86-451-86403826; Email: huying@hit.edu.cn.

[3]# These two authors contributed equally to the work.

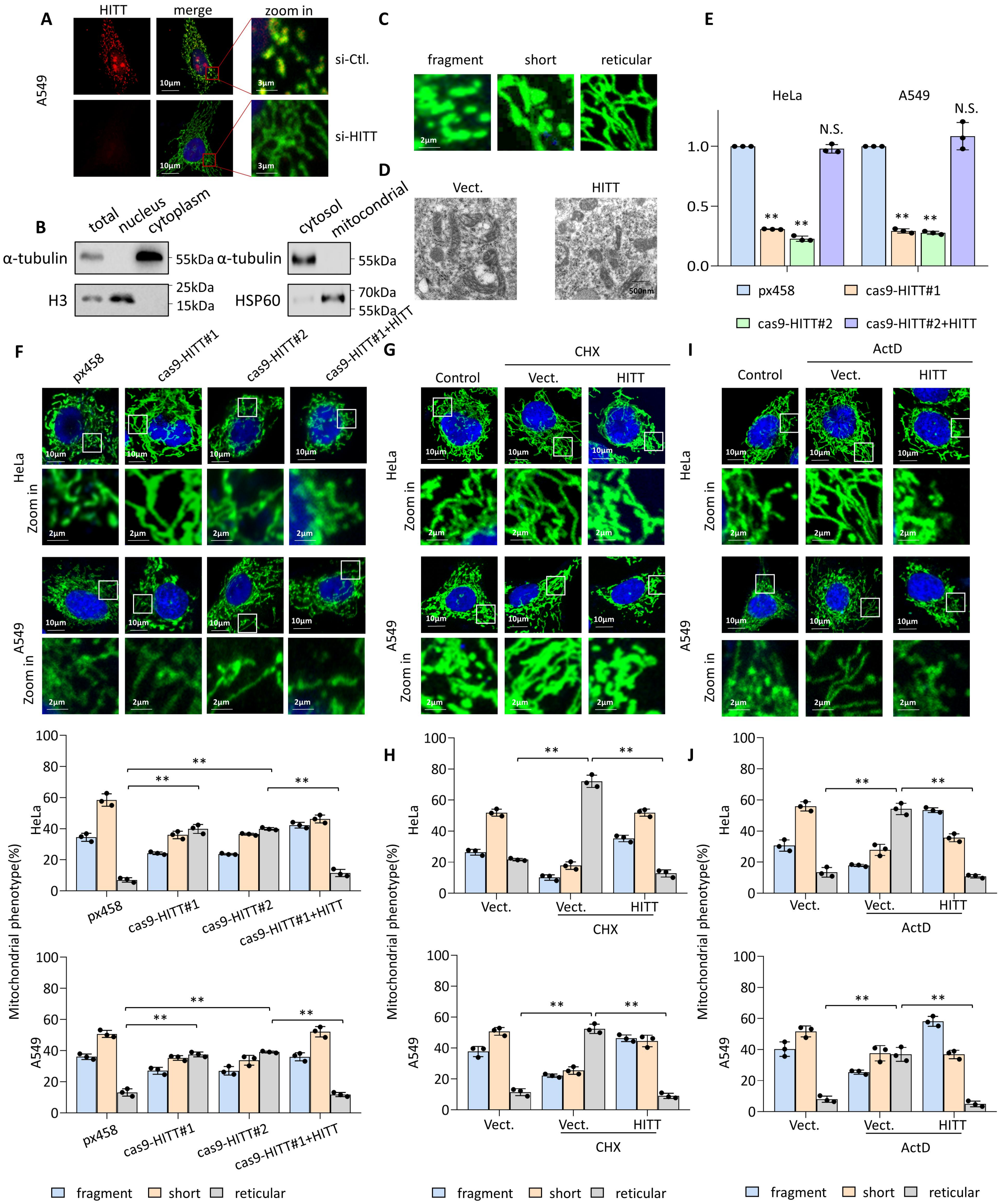
A
HITT
merge
zoom in
A549
si-Ctl.
si-HITT
10μm
3μm
B
total
nucleus
cytoplasm
α-tubulin
55kDa
H3
25kDa
15kDa
cytosol
mitocondrial
α-tubulin
55kDa
HSP60
70kDa
55kDa
C
fragment
short
reticular
2μm
D
Vect.
HITT
500nm
E
HeLa
A549
1.0
0.5
0.0
**
N.S.
px458
cas9-HITT#1
cas9-HITT#2
cas9-HITT#2+HITT
F
px458
cas9-HITT#1
cas9-HITT#2
cas9-HITT#1+HITT
HeLa
Zoom in
A549
Zoom in
G
Control
CHX
Vect.
HITT
I
ActD
Control
Vect.
HITT
H
J
Mitochondrial phenotype(%)
HeLa
A549
0
20
40
60
80
100
Vect.
CHX
ActD
fragment
short
reticular

Figure S1. HITT regulates mitochondrial size

(A) Representative images of RNA-FISH staining of HITT and HSP60 in A549 cells. (B) Representative western blot images of α-tubulin, H3 and HSP60 in cytoplasmic, nuclear, and mitochondrial fraction lysis. (C) Representative confocal images of fragmented (UV treatment for 24 h), short (No treatment) and reticular (UV treatment for 6 h) mitochondrial morphologies determined by immunostaining with anti-HSP60 antibody (green). Bar scale:2 μm. (D) Transmission electron microscopy (TEM) images of mitochondrial morphology in the control and HITT overexpressing HeLa cells. Bar scale: 500nM. (E-F) CRISPR/Cas9-mediated HITT KD or HITT rescue efficiency was confirmed by qRT-PCR in HeLa and A549 cells, respectively(E). 18s was used as an internal control. Representative confocal images of mitochondrial morphology as indicated by immunostaining with anti-HSP60 antibody (green) in CRISPR/Cas9-mediated HITT KD or HITT rescue cells (F, top). Quantification of mitochondria size were presented in the bar graphs (F, bottom). (G-J) Representative confocal images of mitochondrial morphology in the control and HITT overexpressing HeLa and A549 cells by immunostaining with anti-HSP60 antibody (green) in the presence or absence of cycloheximide (CHX, G) and Actinomycin D (I). Nuclear DNA was counter stained with DAPI (4′,6-diamidino-2-phenylindole) in blue. Scale Bar in F,G,I =10 μm. Quantification of mitochondria size were presented in the bar graphs (H and J). Data represented as means ± SEM in the bar graphs. $^{**}P < 0.01$, N.S., not significant.(E,F,H,J) .

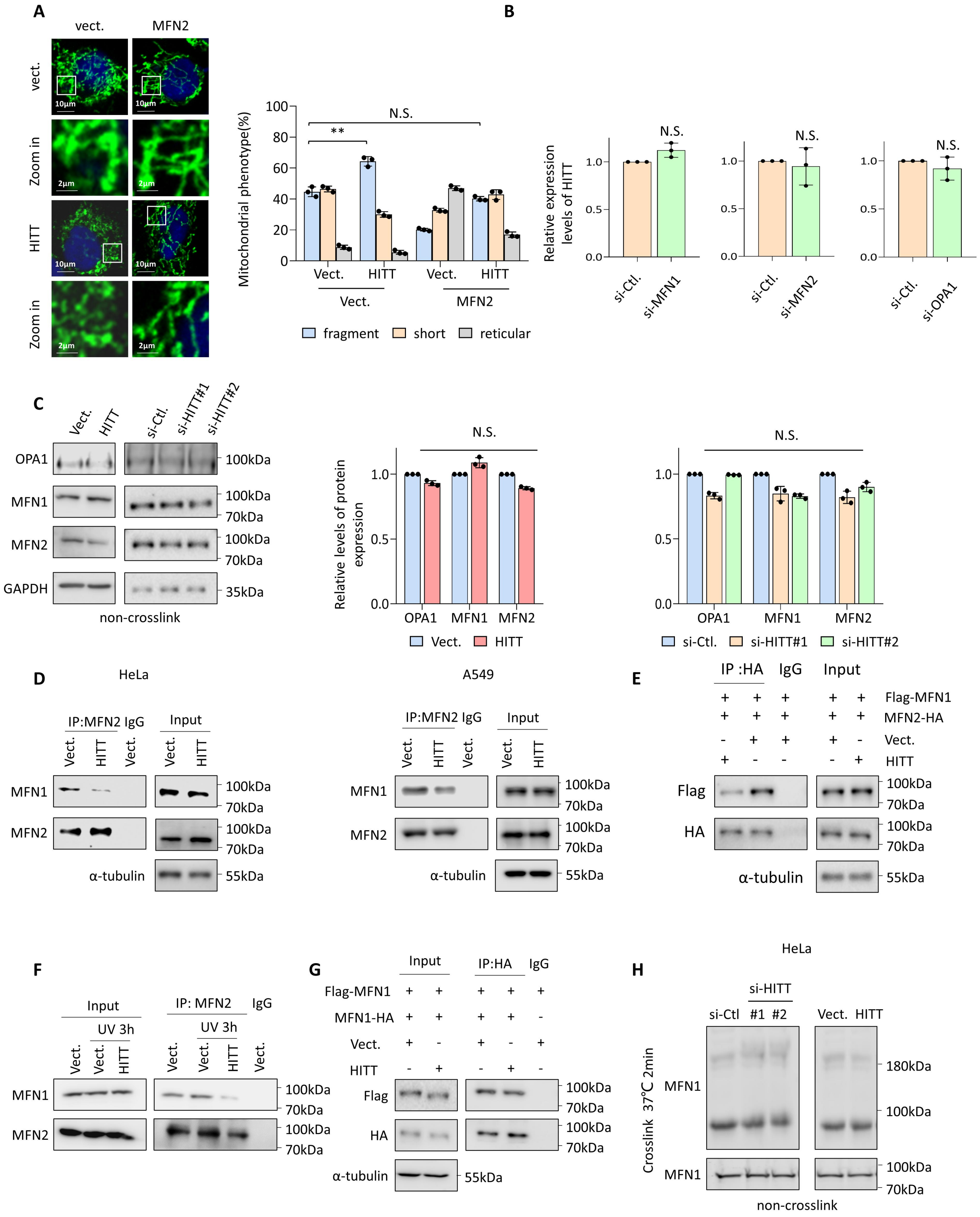

Figure S2. HITT prevents MFN2 homo- and heterotypic complex formation

(A) Representative confocal images of mitochondrial morphology as indicated by immunostaining with anti-HSP60 antibody (green) in the control and HITT overexpression HeLa cells with or without MFN2 overexpression (left). Quantification of mitochondrial morphology were presented in the bar graphs (right). (B) Relative expression levels of HITT were determined by qRT-PCR after DRP1, MFN1 and MFN2 KD in HeLa cells. 18s was used as an internal control. (C) Representative western blot images of DRP1, MFN1 and MFN2 in HITT overexpression and KD HeLa cells. GAPDH act as a loading control (left). Quantification of DRP1, MFN1 and MFN2 expression levels were presented in the bar graphs (right). (D-F)The interaction between endogenous (D) or exogenous (E) MFN1 and MFN2 was determined by a IP assay in the control (D,E) or UV treated (F) control and HITT overexpressing HeLa or A549 cells. IgG was used as a negative control. (G) The MFN1 homotypic dimerization was analyzed by a IP assay after simultaneously overexpressing Flag-MFN1 and MFN1-HA, with or without HITT. IgG was used as a negative control. (H) The relative levels of MFN1 dimers and monomers were analyzed by western blot analysis following glutaraldehyde cross-linking in cell lysates generated from HITT-overexpressing or KD cells. Data represented as means ± SEM in the bar graphs. ***P* < 0.01., N.S., not significant (A-C).

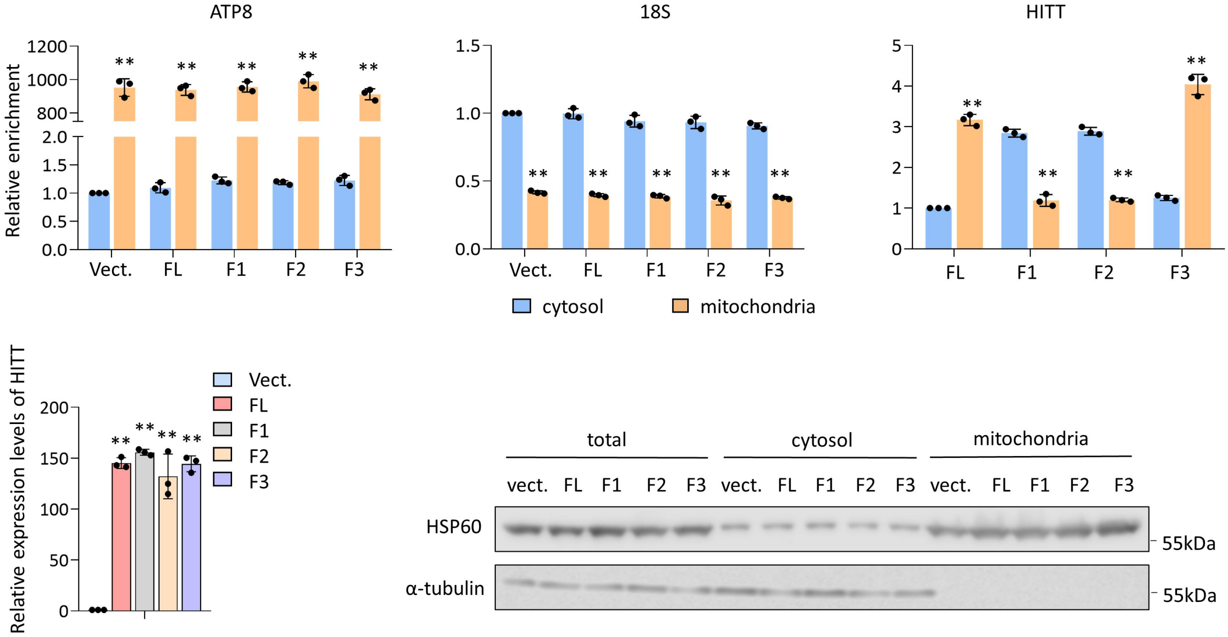

Figure S3. Localization of HITT truncation in cytosol and mitochondria.

The truncated HITT distribution in the cytosol and mitochondria were determined using qRT-PCR after fractionation. ATP8 and 18S were used as mitochondrial and cytosol markers, respectively. HITT fragments transfect efficiencies were measured by qRT-PCR. Representative western blot images of α-tubulin and HSP60 in cytosol and mitochondrial fraction lysis. Data represented as means ± SEM in the bar graphs. $^{**}P <$ 0.01.

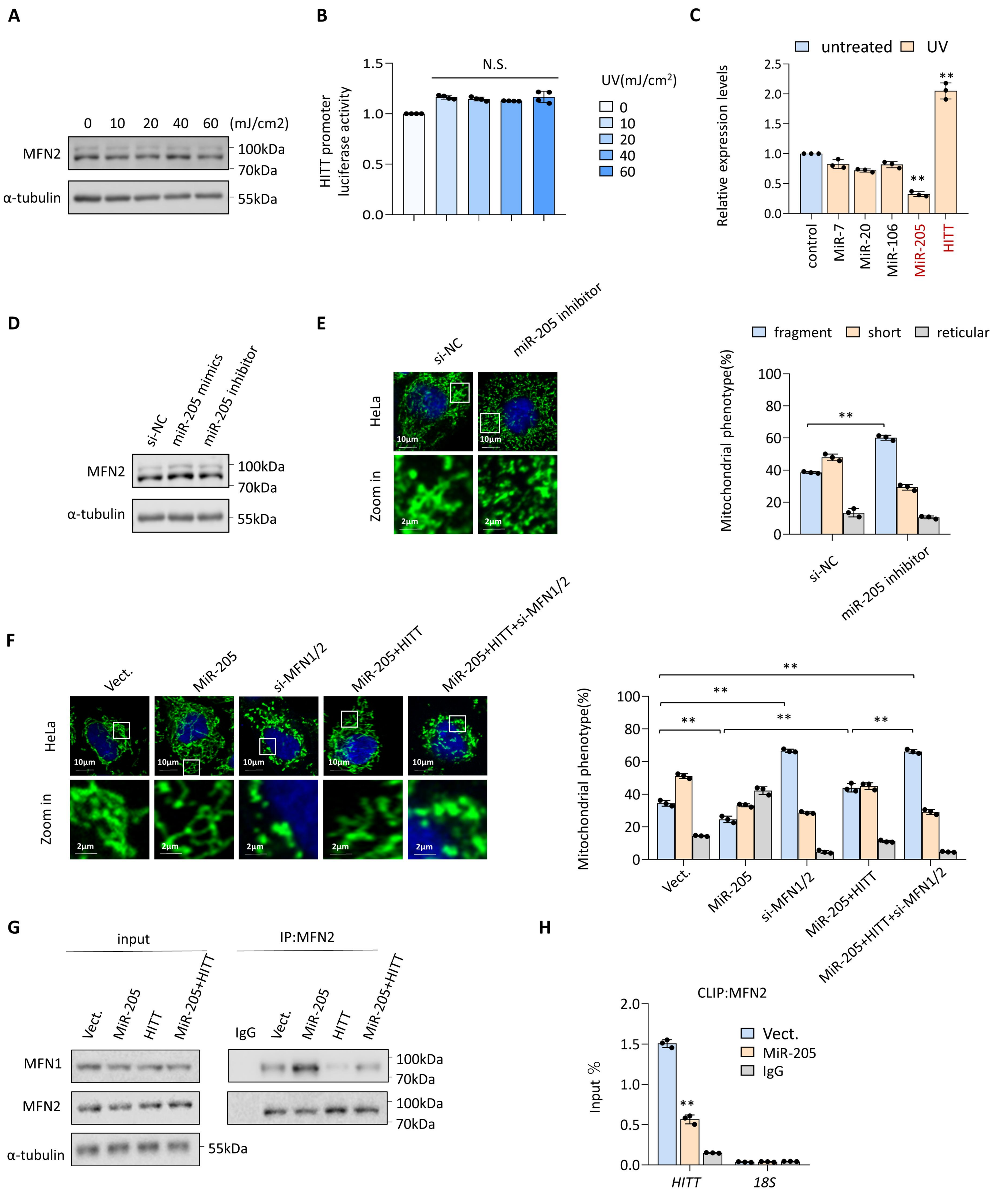

Figure S4. HITT expression is suppressed by miR-205 in stressed cancer cells

(A) The expression levels of MFN2 were determined by western blot after UV treatment in HeLa cells. (B) The promoter luciferase activity of HITT was measured in HeLa cells after UV (0, 10, 20, 40, 60 mJ/cm2) treatments.

(C) The expression levels of HITT and microRNAs were determined by qRT-PCR after UV treatment in HeLa cells. (D) The expression levels of MFN2 were determined by western blot after MiR-205 overexpression and KD. (E-F) Representative confocal images of mitochondrial morphology as indicated by immunostaining with anti-HSP60 antibody (green) in MiR-205 KD HeLa cells (E,left), and in HITT overexpression or MFN1/MFN2 KD cells with or without MiR-205 overexpression(F,left). Quantification of mitochondrial morphology were presented in the bar graphs (E-F,right). (G) The interaction between MFN1 and MFN2 was determined by a IP assay in MiR-205 overexpression cells with or without HITT overexpression in HeLa cells. IgG was used as a negative control. (H) MFN2 associated HITT was measured by CLIP MFN2 after MiR-205 overexpression in HeLa cells. 18S mRNA and CLIP IgG were used as negative controls. Data represented as means ± SEM in the bar graphs. ***P* < 0.01. N.S., not significant. (B,C,E,F,H).

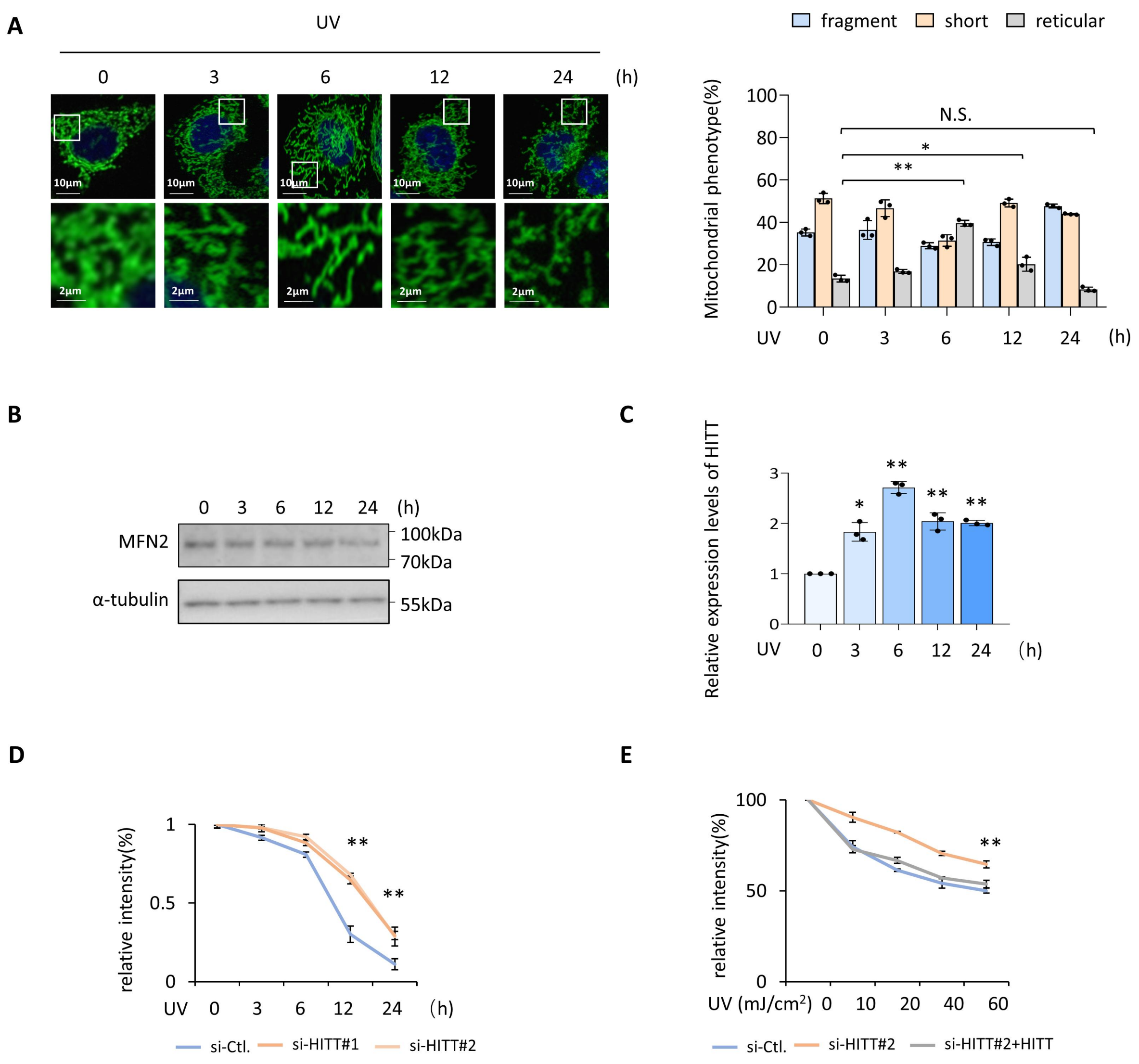

Figure S5. The HITT regulates UV-induced adaptive survival in cancer cells

(A) Representative confocal images of mitochondrial morphology as indicated by immunostaining with anti-HSP60 antibody (green) in 40 mJ/cm2 UV treated and recover for 0, 3, 6, 12, and 24 hours HeLa cells (left). Quantification of mitochondrial morphology were presented in the bar graphs (right). (B-C) The expression levels of MFN2 (B) or HITT (C) were determined by western blot or qRT-PCR after UV treatment in HeLa cells. (D-E) Cell survival rates were determined by crystal violet staining assays after KD HITT with(E) or without HITT(D) exprerssion cells under indicated treatment. Data represented as means ± SEM in the bar graphs. **P < 0.01. *P < 0.05. N.S., not significant. (A,C,D,E).

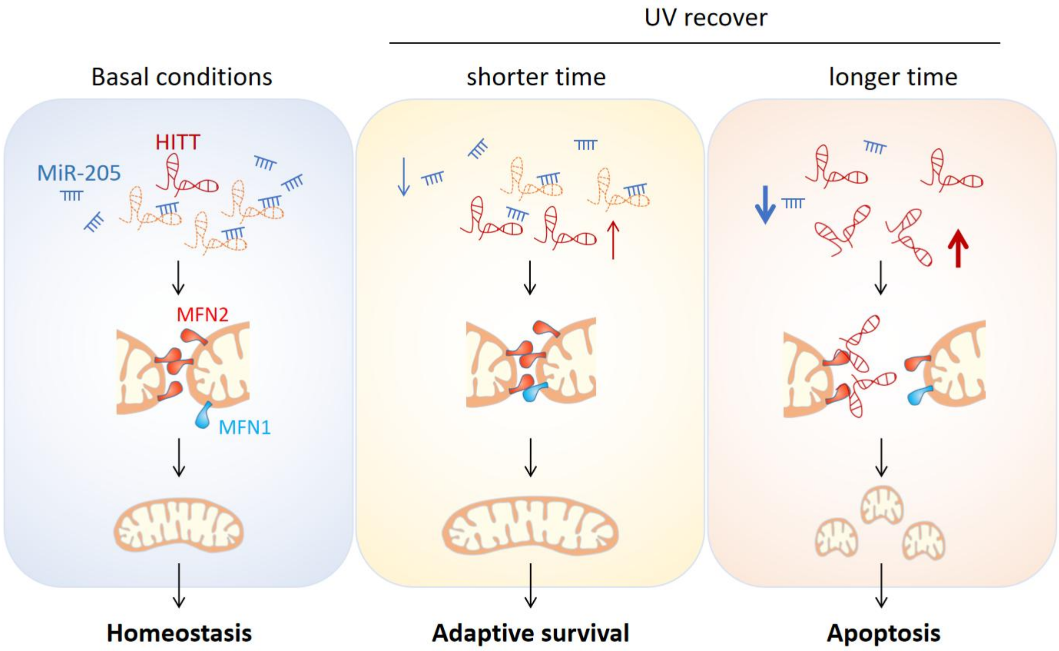

Figure S6. Proposed model of MiR-205/HITT/MFN2 axis in UV-induced stress

Under unstressed conditions, HITT levels are limited by the expression of MiR-205, Under UV-induced stress, MFN2 mediates mitochondrial fusion facilitates adaptive survival. When the stress is enhanced, HITT stability increases as a consequence of MiR-205 downregulation. As such, HITT directly binds MFN2 and disturbs MFN2 homo- or heterotypic complex formation, attenuating mitochondrial fusion and leading to apoptosis.

Supplementary Table S1-Primers and probes used in this study

Name	Forword sequence	Reverse sequence
Flag-MFN1	AAGGGTACCGAGCTCGGATCC<br>GCAGAACCTGTTTCTCCACTGAA	CGCGGGCCCTCTAGACTCGAG<br>TTAGGATTCTTCATTGCTTGAAGGT
Flag-MFN2	AAGGGTACCGAGCTCGGATCC<br>TCCCTGCTCTTCTCTCGATGCAA	CGCGGGCCCTCTAGACTCGAGTTA<br>TCTGCTGGGCTGCAGGTA
GST-MFN1	CCCCTGGGATCCCCGGAATTC<br>GCAGAACCTGTTTCTCCACTGAA	CTCGAGTCGACCCGGGAATTC<br>TTAGGATTCTTCATTGCTTGAAGGT
GST-MFN2	CCCCTGGGATCCCCGGAATTC<br>TCCCTGCTCTTCTCTCGATGCAA	CTCGAGTCGACCCGGGAATTCTTA<br>TCTGCTGGGCTGCAGGTA
GST-MFN2(1-320)	CCCCTGGGATCCCCGGAATTC<br>TCCCTGCTCTTCTCTCGATGC	CTCGAGTCGACCCGGGAATTC<br>TTACATGCCCTGGGCTTTCTGAA
GST-MFN2(321-500)	CCCCTGGGATCCCCGGAATTC<br>CCTGAAGGAGGGGGCGC	CTCGAGTCGACCCGGGAATTC<br>TTAGCCATCTATCATGTCCTGCTG
GST-MFN2(501-650)	CCCCTGGGATCCCCGGAATTC<br>TTGAAACCCCTCCTTCCTGTGT	CTCGAGTCGACCCGGGAATTC<br>TTACAGACGCTCATAGACGTAGAGG
GST-MFN2(651-754)	CCCCTGGGATCCCCGGAATTC<br>ACCTGGACCACCAAGGCCA	CTCGAGTCGACCCGGGAATTC<br>CTATCTGCTGGGCTGCAGGTAC
HITT	ACACAAATGCTGGCCTCTGTCA	GGCAAGTGGCAAAGCCTCTC
18s	AACTTTCGATGGTAGTCGCCG	CCTTGGATGTGGTAGCCGTTT
GAPDH	TCGTCTGAGGGGACAGGAGGATC	GGAAAGGCAAGTCCAGAGGTGGG
MiR-205	ACACTCCAGCTGGGTCCTTCATTCCACCGG	TGGTGTCGTGGAGTCG
HITT(1-124)	GGTCCCTGTCCTCACAGAGTT	TGTTCTTGTCTATCGTCTTCTTGC
HITT(125-1029)	AAGGAGGGTAGGAGTCTTGGTC	CCGAAATAAAGGCAGGAGTGA
HITT(1030-2050)	CCAAAAGGCAAAAGCAGGGTG	CAGGAAGGCTCTGGCTCAGGAAT
MALAT1	GAATTGCGTCATTTAAAGCCTAGTT	GTTTCATCCTACCACTCCCAATTAAT
ATP8	ACCCCCATACTCCTTACACT	TGAGGGAGGTAGGTGGTAGTT
U6	CTCGCTTCGGCAGCACA	AACGCTTCACGAATTTGCGT
HITT probe-1	TGAAAGACCAGTACAGTGTGTG	
HITT probe-2	TAGCACGGAGGACGCAAGAAAG	
HITT probe-3	GAAATAAAGGCAGGAGTGAGCG	
Targeting exon 3 *HITT*-BbsI	CACCGGGAGGGGCACGGTAACACC	AAACGGTGTTACCGTGCCCCTCCC
Targeting full-length *HITT*-BbsI	CACCGGTACCCTCGCTCTCAGCGG	AAACCCGCTGAGAGCGAGGGTACC
Targeting full-length or exon 3 *HITT*-BsaI	CCGGTGCCAGACGGGTCGGGTG	AAACCACCCGACCCGTCTGGCA
